# A user-friendly tool to transform large scale administrative data into wide table format using a mapreduce program with a pig latin based script

**DOI:** 10.1186/1472-6947-12-151

**Published:** 2012-12-22

**Authors:** Hiromasa Horiguchi, Hideo Yasunaga, Hideki Hashimoto, Kazuhiko Ohe

**Affiliations:** 1 Department of Health Management and Policy, Graduate School of Medicine, The University of Tokyo, 7-3-1 Hongo, Bunkyo-ku, Tokyo, 1138555, Japan; 2 Department of Health Economics and Epidemiology Research, School of Public Health, The University of Tokyo, Tokyo, Japan; 3 Department of Medical Informatics and Economics, Graduate School of Medicine, The University of Tokyo, Tokyo, Japan

**Keywords:** MapReduce, Pig Latin, Large scale administrative data, User-defined functions

## Abstract

**Background:**

Secondary use of large scale administrative data is increasingly popular in health services and clinical research, where a user-friendly tool for data management is in great demand. MapReduce technology such as Hadoop is a promising tool for this purpose, though its use has been limited by the lack of user-friendly functions for transforming large scale data into wide table format, where each subject is represented by one row, for use in health services and clinical research. Since the original specification of Pig provides very few functions for column field management, we have developed a novel system called *GroupFilterFormat* to handle the definition of field and data content based on a Pig Latin script. We have also developed, as an open-source project, several user-defined functions to transform the table format using *GroupFilterFormat* and to deal with processing that considers date conditions.

**Results:**

Having prepared dummy discharge summary data for 2.3 million inpatients and medical activity log data for 950 million events, we used the Elastic Compute Cloud environment provided by Amazon Inc. to execute processing speed and scaling benchmarks. In the speed benchmark test, the response time was significantly reduced and a linear relationship was observed between the quantity of data and processing time in both a small and a very large dataset. The scaling benchmark test showed clear scalability. In our system, doubling the number of nodes resulted in a 47% decrease in processing time.

**Conclusions:**

Our newly developed system is widely accessible as an open resource. This system is very simple and easy to use for researchers who are accustomed to using declarative command syntax for commercial statistical software and Structured Query Language. Although our system needs further sophistication to allow more flexibility in scripts and to improve efficiency in data processing, it shows promise in facilitating the application of MapReduce technology to efficient data processing with large scale administrative data in health services and clinical research.

## Background

Secondary large scale data such as nation-wide administrative data are increasingly utilized in clinical and health service research for timely outcomes studies in real world settings [1-5]. This trend has further been fueled by recent improvements in informatics technology for handling ultra large volumes of on-site data through work parallelization and cloud computing. For example, the launch of the Sentinel System by the US Food and Drug Administration in 2008 aimed at establishing an active surveillance system for monitoring drug safety in real-time, using electronic data from multiple healthcare information holders. In Japan, the Diagnosis Procedure Combination (DPC) inpatient database survey has collected nationwide administrative data since 2003 [6], and several epidemiological studies have been based on this inpatient database [7-12].

**Figure 1:**
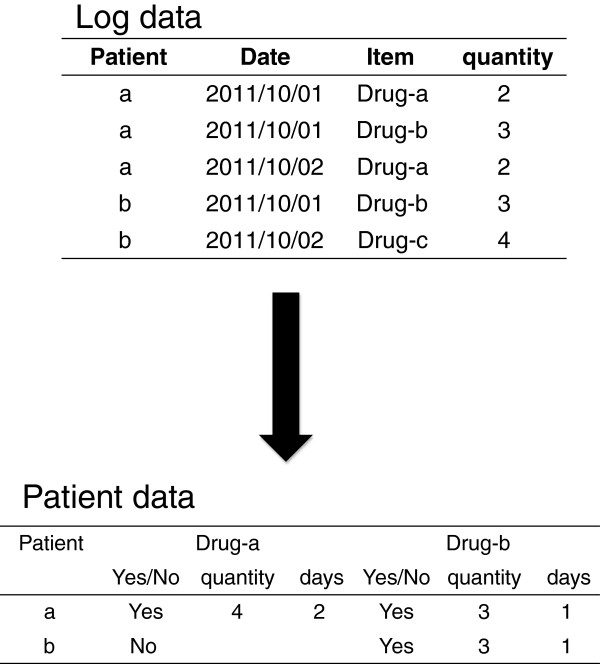
Data transformation from log data to a wide table.

Administrative data including time (e.g., day, hour), procedure, or episode (e.g., hospitalization or visit), are often presented in long table format. However, prevalent statistical software for epidemiological analyses prefers datasets prepared in wide table format with each individual record corresponding to one row. Extracting data from different sources requires linkage of data with multiple unique patient identifiers, and complicated steps for data merge and transformation (Figure 1). Furthermore, it is often necessary in epidemiological studies to calculate the time interval between different events recorded in different rows, and then to transform these data into a wide table column. Suppose one wished to know whether administration of antibiotics within three days after surgery reduced the chance of postsurgical infection. Then, the time interval between the first and last dates of antibiotic administration would need to be calculated and queried.

Data processing and transformation as described above can be simply handled using existing Structured Query Language (SQL) technology [13] with a moderate sized dataset. However, treatment of ultra large databases such as the nationwide administrative data is beyond the scaling capacity of SQL, and instead parallelized environments are required. Open source MapReduce technology such as Hadoop has become popular as a software framework for large scale distributed data analysis [14,15]. The usefulness of Hadoop, however, is still limited. Many epidemiologists, health service researchers, and health policy analysts who are familiar with the declarative style of existing statistical software and SQL commands do not use Hadoop because MapReduce programming is too rigid and difficult for these end users to write procedural code. Also, these researchers often need trial-and-error ad-hoc analysis for data description and planning optimal analytic strategy. For such purposes, we need a more user-friendly framework that allows iterative, easy, and quick transformation of ultra-large scale administrative data into an analytic dataset. Recent development of the Pig Latin language is promising for filling this gap between procedural programmers and data users, and allows user-friendly use of MapReduce technology, though its use in the bioinformatics arena is still limited [16].

Given the background and incentives above, we have developed several user-defined functions (UDF) to process large scale administrative data for ease of epidemiological analysis, based on a Pig Latin script in the Hadoop framework [17]. The developed script is very easy to use even for researchers who are accustomed to using declarative command syntax for statistical software and SQL. The developed functions were tested with a large claims database for response speed and scalability and, as presented below, achieved fairly satisfying results.

## Implementation

### Referred technologies

**Figure 2:**
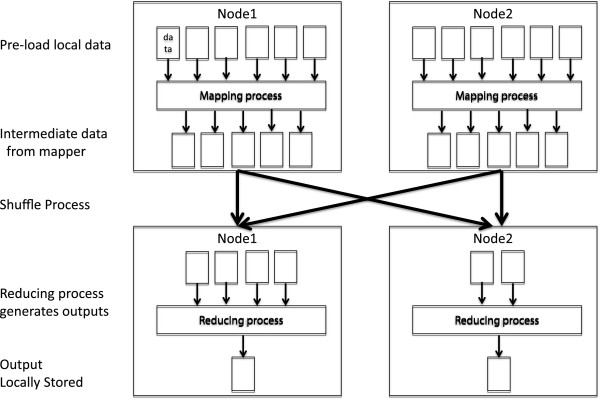
Map Reduce architecture.

Map and Reduce are common to many functional programming languages such as Lisp and Scheme. Google recently popularized the use of Map and Reduce as a simpler solution for parallelizing computation [18] for a certain subset of problems compared to other approaches such as Parallel Virtual Machines [19] (Figure 2). One major benefit of the MapReduce approach is the ability to focus solely on the computation, and not the shuffling of data between processors. The programmer only needs to consider the computation itself and can assume that the data will be available as required. This allows users with some programming experience to create and run jobs without extensive training in parallel computing. The second major benefit of MapReduce concerns data locality. With the MapReduce paradigm, most of the computation is done on a slave node, which contains a copy of the input data. This requires the minimal amount of data being sent over the network, resulting in increased overall efficiency.

Hadoop is an open-source implementation of the MapReduce parallel programming paradigm and is supported by the open-source community. Hadoop provides both the MapReduce parallel computation framework and a distributed file system (called the Hadoop Distributed File System, HDFS). Hadoop, which is an Apache Foundation project written in Java, provides a master–slave architecture where a single master node coordinates many slave machines, which carry out data storage and the actual computation. To enable data-local processing, each slave machine tries to use only data stored on the same machine for computation. This requires very little shuffling of data over the network, resulting in decreased demand for network I/O bandwidth. Additional slave nodes can be added to the cluster to increase HDFS storage capacity and computational power as necessary.

Finally, Apache Pig is a platform for analyzing large datasets and consists of a high-level language for expressing data analysis programs, coupled with the infrastructure for evaluating these programs. The salient property of Pig programs is that their structure is amenable to substantial parallelization, which in turn enables them to handle very large datasets [17,20]. Pig’s infrastructure layer consists of a compiler on the user’s client machine that transforms Pig Latin programs into sequences of MapReduce programs that run in parallel on the nodes of a Hadoop cluster. Pig is a Java client side application installed locally by users, and thus nothing is altered on the Hadoop cluster itself [16].

Despite its innovativeness and broader feasibility, the original Pig Latin has several drawbacks. Firstly, it has only a “join” script to transform log data into a wide table format, in which the data are compiled for one field at a time. This prevents processing multiple fields in parallel, resulting in slow processing and inefficient script formation. Secondly, Pig includes only very poor functions for date processing. It is quite cumbersome to use Pig for filtering data by date or calculating day intervals between events. These drawbacks must be overcome to make Pig based scripts suitable for epidemiological studies.

### UDFs for data transformation into a table format

Transformation of long-shaped log data into a wide table format requires management of the column field scheme, e.g., assignment of column names and field locations, and definition of the data content. Since the original Pig has very limited functions for column field management, we newly developed *GroupFilterFormat* to handle the definition of field and data content. *GroupFilterFormat* also provides information linkage between different code systems, and generates new categories and values. For example, pharmaceutical codes (such as Universal Product Numbers in the United States or Japanese Article Numbers for pharmaceuticals in Japan) by product may be cumbersome to handle, and one may wish to categorize them into larger groups of pharmaceutically equivalent products (according to their generic name). Furthermore, suppose pharmaceutical codes correspond not only to the types of medication, but also to the dose of the medication. *GroupFilterFormat* defines which pharmaceutical code should be categorized into a new larger category, and attaches the numeric dose information to the code.

The input format for *GroupFilterFormat* is as follows: 'groupname (item1 [value1, value2 …], item2 … ), … 'where groupname = a new group name corresponding to a new field in wide format, item = the original code per item, and value = a numeric value attached to each item.

**Figure 3:**
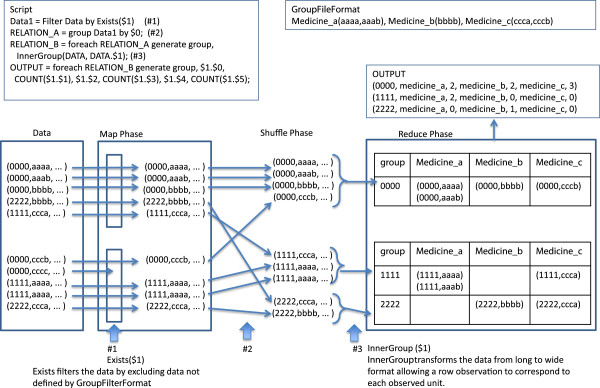
**Step-by-step example of data transformation.** $ *N* indicates the *N*th column in the data field.

In the Map phase, Exists filters the data by excluding data not defined by GroupFilterFormat, and reduces the data volume to improve the efficiency of data processing. In the Reduce phase, InnerGroup transforms the data from long to wide format allowing a row observation to correspond to each observed unit, e.g., patient or admission event. The original Pig has no functions for column management. Developed data in a table format are prepared for numeric processing. Value-Join provides quantitative values linked with qualitative categorical information as defined in GroupFilterFormat for further numeric processing. For numeric calculation, the calculation functions originally available in Pig can be used (e.g., COUNT, SUM, MAX, and so on) (Figure 3).

### UDFs for the management of date data

**Figure 4:**
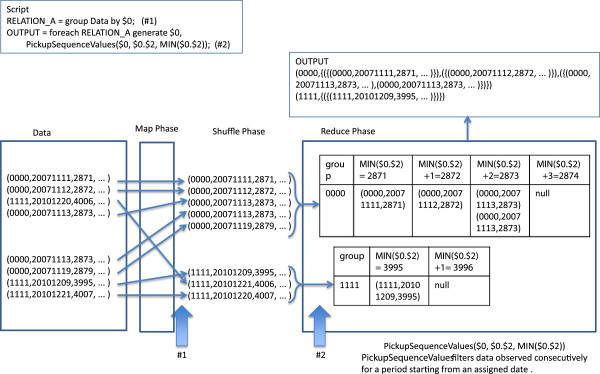
**Step-by-step example of date management.** $ *N* indicates the *N*th column in the data field.

*GetDaySpan* calculates a day interval between two dates. *AddDaySpan* adds an *n* day interval to a date to obtain the date after the interval. These two UDFs are useful for calculating age and event intervals. *PickupSequenceValues* filters data observed consecutively for a period starting from an assigned date. This UDF is useful for extracting log data of pharmaceutical administration repeated over a period (Figure 4).

### Benchmark environment and test dataset

Time efficiency is an important issue in data management. The main goal of this study was to provide researchers with open-source, time-efficient software for handling large scale administrative data. Existing methods designed to handle small datasets would require a vast amount of time to process a large dataset. This is a serious problem because it may hinder researchers in carrying out large-data studies. We developed our software to solve this problem and contribute to the enhancement of research using a large administrative database. Consequently, we evaluated the performance of the software mainly in terms of time efficiency and scalability.

The Elastic Compute Cloud (EC2) infrastructure service from Amazon was used as a test bed for the performance evaluation. We adopted a Large Instance provided by Amazon EC2 with the following configuration: 7.5 GB memory, 4 EC2 Compute Units (2 virtual cores with 2 EC2 Compute Units each), 850 GB instance storage, and a 64-bit platform.

In this benchmarking test, we created dummy administrative data for in-hospital services containing patient discharge summary data and medical activity logs for 20 different kinds of medications. We prepared discharge summary data for 2.3 million inpatients and medical activity log data for 950 million events. The Input and Output data image is as shown in Additional file 1: Appendix 1 while the program script used for the benchmark test is given in Additional file 2: Appendix 2.

We created a Hadoop cluster on Amazon EC2, composed of one master for the master name and job tracker node, and varying numbers of slave nodes for task tracker and data nodes. For the processing speed benchmark, we used varying sized subsamples of the benchmark test data, that is, 1/1 sample (23 million patients), 1/2 sample (11.5 million patients), 1/4 sample (5.7 million patients), and 1/8 sample (2.6 million patients), and ran the same script 20 times with each subsample to measure the processing time using one master node and 4 slave nodes. For the scaling benchmark, we used the entire sample data, and ran the same script 20 times using one master node and 2 slave nodes. Then we doubled the number of slave nodes until 48 nodes were used, repeatedly measuring the processing time.

## Results and discussion

**Figure 5:**
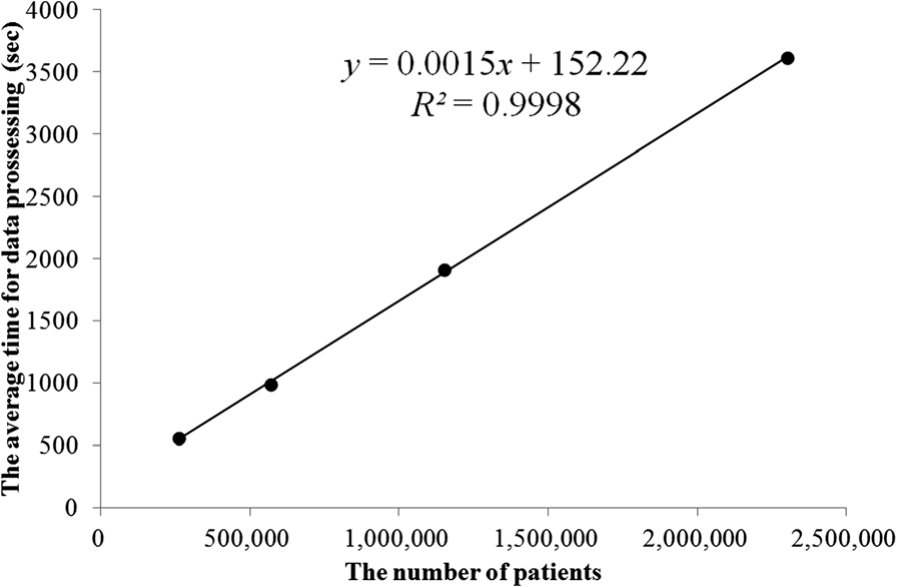
**Processing speed benchmark.** Dots indicate the average processing time for 20 trials. The line indicates the prediction equation fitted with a linear regression.

Table 1 and Figure 5 present the results of the processing speed benchmark using regression in a linear model, (time(sec)) = 0.0015*(record)+155.22 R2=0.9998. As shown in the graph, there is a clear linear relationship between the processing time and data size. The intercept of the model is significant, at 155 seconds, which should correspond to the lead time for batch processing.

**Table 1:** Results of the processing speed benchmark

**Number of records**	**Average processing time (s)**	**Max time (s)**	**Min time (s)**
261,369	554.5976	565.765	545.513
569,738	986.0611	1000.902	971.052
1,150,684	1911.162	1932.173	1890.286
2,301,367	3616.403	3,673.40	3,598.07

**Figure 6:**
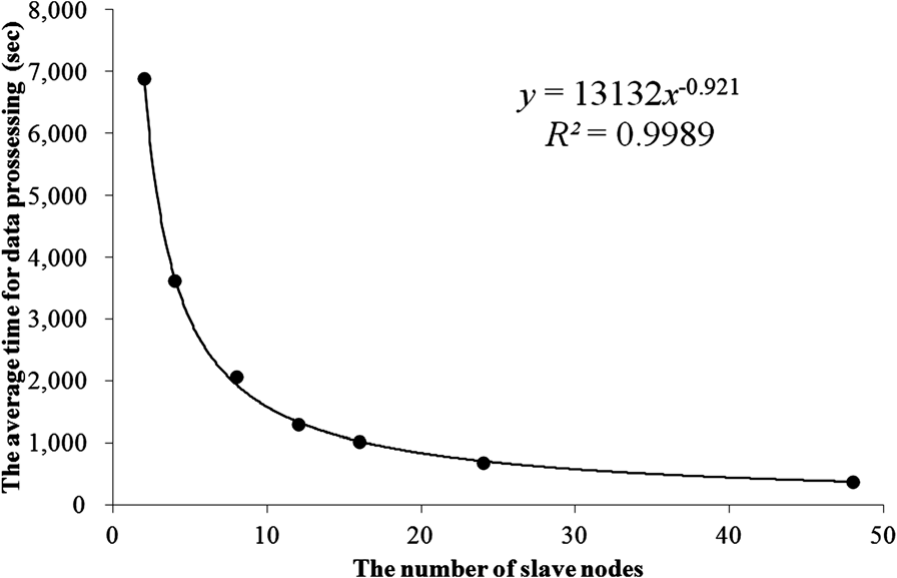
**Scaling benchmark.** Dots indicate the average processing time for 20 trials. The line indicates the prediction equation fitted with a power regression.

Table 2 and Figure 6 give the results of the scaling benchmark using regression in a power model, (time(sec)) = 13132(number of node)^-0.921^ R2=0.9989. The intercept of the model is significant and this result shows that using double the number of nodes reduces processing time by 47%.

**Table 2:** Results of the scalability benchmark

**Number of slave nodes**	**Average processing time (s)**	**Max time (s)**	**Min time (s)**
2	6,892.868	6,986.503	6,844.374
4	3,616.403	3,673.398	3,598.065
8	2,063.208	2,087.145	2,037.378
12	1,301.092	1,326.391	1,280.319
16	1,022.917	1,133.464	985.958
24	677.832	690.765	670.458
48	379.049	401.013	370.314

The motivation for developing the current system was to simplify the use of large scale administrative databases in epidemiological and health service research, and for policy evaluation. We believe the developed system will be useful and will contribute to the above goal for the following reasons.

Firstly, the developed system achieves satisfying scaling for conversion of a large scale dataset for parallelization with Hadoop. Because of the overhead of managing each node, adding additional nodes yields a diminished volume of transactions, but retains adequate scaling ability. Processing the 950 million log entries for administrative activities in the performance test took one hour at a cost of 10 US dollars using a parallel environment such as the Amazon system with one master and eight slave nodes. To complete the task in 10 minutes requires one master plus 48 slave nodes at a cost of about 50 US dollars. Thus, the system allows users to choose between the tradeoff of time response and cost.

Secondly, the current system uses only free and open-source components. It uses the Hadoop framework for distributed data processing, and the Pig Latin language for script development. These are free-share open-source software (OSS) products under the Apache License. System development of the UDFs in the current study is also an OSS project, freely available for use and alteration. Furthermore, Pig can execute the same script on local computers even without Hadoop. Durok is a subproduct that has developed from this use of Pig, allowing the currently developed system to be used as original application software. At present, Durok is open-source software available under an Apache License; however, it is not an official project of the Apache Foundation. The Durok system can be applied to small datasets that can be processed without a distributed data processing environment.

Finally, the system achieved quick response in processing the large administrative database to allow convenient ad-hoc analysis in a trial-and-error fashion. Quick and easy access to large databases allows researchers and analysts broader opportunities for investigating innovative research questions, generating hypotheses to be tested in formal research, and ad-hoc monitoring of adverse events.

The current system still needs further development of the UDFs to allow more complicated data transformation with simpler scripts. Currently, the proposed UDFs are functionally separated into grouping and date functions owing to restrictions in the format design of *GroupFilterFormat.* However, users may wish to identify patterns in timing and types of administered pharmaceuticals through data mining to find best practice patterns in a real setting. To satisfy such requirements, the format design needs further development to allow flexibility in setting a reference time point in *GroupFilterFormat.*

We believe that the present system is generalizable to any large scale administrative database which has a similar data format to the DPC data. Another challenge is to further improve efficiency in data processing with increased data sizes. The Reduce process is a limiting factor in improving the speed of data processing. Currently the proposed scheme needs two iterations of the Reduce step to transform a table (by *Innergroup*) and numeric calculation. How to decrease the number of Reduce processes will be the key to achieving further speedup. This may be possible by developing original UDFs for numeric processing, or by reordering data processing to avoid the second Reduce step.

## Conclusions

Using a MapReduce program with a Pig Latin-based script, we developed a tool to transform ultra large administrative data into a wide table format. This tool is very simple and easy to use by researchers, and shows promise in applying MapReduce technology to efficient data processing in health services and clinical research with large scale administrative data.

## Availability and requirements

• Project name: The University of Tokyo DPC project

• Project home page: http://github.com/hiromasah/charsiu

• Operating system(s): Platform independent

• Programming language: Java

• Other requirements: Java 1.3.1 or higher, Hadoop 1.0.0, Pig 0.9.2

• License: Apache

• Any restrictions to use by non-academics: This is an open source project.

## Abbreviations

DPC: Diagnosis Procedure Combination; SQL: Structured Query Language; UDF: User-defined functions; HDFS: Hadoop Distributed File System; EC2: Elastic Compute Cloud; OSS: Open-source software.

## Competing interests

The authors have no competing interests.

## Authors’ contributions

HH1 conceived the idea for the tool and evaluation, designed the development of the tool, ran the evaluation, and drafted the manuscript. HY and HH2 participated in the design of the tool. HY, HH2, and KO critically revised the draft in terms of important intellectual content. All authors read and approved the final manuscript.

## Pre-publication history

The pre-publication history for this paper can be accessed here:

http://www.biomedcentral.com/1472-6947/12/151/prepub

## Supplementary Material

Additional file 1 **Appendix 1. **Dataset format. Click here for file

Additional file 2 **Appendix 2. **Pig script. Click here for file
