# Supplementary material for: A user-friendly tool to transform large scale administrative data into wide table format using a mapreduce program with a pig latin based script
Source: BMC Med Inform Decis Mak. 2012 Dec 22;12:151. doi: 10.1186/1472-6947-12-151 (PMC3545829; doi:10.1186/1472-6947-12-151)
Supplement: Additional file 1 — Appendix 1. Dataset format. [file 1472-6947-12-151-S1.docx]

**Appendix 1. Dataset format**

| Description | Data format |
| --- | --- |
| hospital code | chararray |
| patient code | chararray |
| discharge date | chararray |
| admission date | chararray |
| Data type ex:Drug, Operaton | chararray |
| sequence id1 | chararray |
| sequence id1 | chararray |
| event code for intarnal use | chararray |
| event code for recept | chararray |
| claim code for Japanese Payment system | chararray |
| event name | chararray |
| Quantity | float |
| Unit | chararray |
| cost data | float |
| cost data(drug) | float |
| cost data(material) | float |
| payment rate | chararray |
| charge data | float |
| Fee for service or not | chararray |

Output data format

| Field description | data format |
| --- | --- |
| patient code | chararray |
| amission date | chararray |
| Name of Drug #1 | chararray |
| First day of Delivery | chararray |
| Last day of Deliverly | chararray |
| Name of Drug #2 | chararray |
| First day of Delivery | chararray |
| Last day of Deliverly | chararray |
| (repeat) |  |
| Name of Drug #32 | chararray |
| First day of Delivery | chararray |
| Last day of Deliverly | chararray |

Drug list

| #1 | aspirin |
| --- | --- |
| #2 | enoxaparin sodium |
| #3 | fondaparinux sodium |
| #4 | dalteparin sodium |
| #5 | heparin calcium |
| #6 | heparin sodium |
| #7 | warfarin potassium |
| #8 | adrenaline |
| #9 | noradrenaline |
| #10 | dopamine hydrochloride |
| #11 | dobutamine hydrochloride |
| #12 | freeze-dried sulfonated human normal immunoglobulin |
| #13 | freeze-dried pepsin treated human normal immunoglobulin |
| #14 | polyethyleneglycol treated human normal immunoglobulin |
| #15 | human normal immunoglobulin |
| #16 | freeze-dried pH4 treated human normal immunoglobulin |
| #17 | freeze-dried ion-exchange-resin treated human normal immunoglobulin |
| #18 | pH4 treated acidic human normal immunoglobulin |
| #19 | polyethyleneglycol treated human normal immunoglobulin |
| #20 | gabexate mesilate |
| #21 | nafamostat mesilate |
| #22 | ulinastatin |
| #23 | vancomycin hydrochloride |
| #24 | vancomycin hydrochloride |
| #25 | arbekacin sulfate |
| #26 | teicoplanin |
| #27 | quinupristin･dalfopristin |
| #28 | linezolid |
| #29 | dioctyl sodium sulfosuccinate･casanthranol |
| #30 | sodium picosulfate hydrate |
| #31 | magnesium citrate |
| #32 | magnesium citrate |
| #33 | sodium･potassium combined drug |
| #34 | monobasic sodium phosphate monohydrate･dibasic sodium phosphate anhydrous |
