# Supplementary material for: A user-friendly tool to transform large scale administrative data into wide table format using a mapreduce program with a pig latin based script
Source: BMC Med Inform Decis Mak. 2012 Dec 22;12:151. doi: 10.1186/1472-6947-12-151 (PMC3545829; doi:10.1186/1472-6947-12-151)
Supplement: Additional file 2 — Appendix 2. Pig script. [file 1472-6947-12-151-S2.docx]

**Appendix 2. Pig script**

%declare GFF 'A300(190024310,190024410,190024510,190074510,190110210,190110310,190110410,190110510,190110610,190110710,190110810,190110910,190111070,190128610,190128710,190128870,190128910,190129010,190129110,190129210,193000110,193000210,193000310,193000410,193000510,193000610,193000710,193000810,193000910,193001010,193001110,193001210,193001370,193003310,193003410,193003510,193003610,193003710,193003810,193005170,193300110,193300210,193300310,193300410,193300510,193300610,193300710,193300810,193300910,193301010,193301110,193301210,193301370,193304310,193304410,193304510,193304610,193304710,193304810,193306370,193500110,193500210,193500310,193500410,193500510,193500610,193500710,193500810,193500910,193501010,193501110,193501210,193501370,193504610,193504710,193504810,193504910,193505010,193505110,193507070),A301(190024610,190074610,190116310,190116410,193001410,193001510,193001610,193001710,193301410,193301510,193301610,193301710,193501410,193501510,193501610,193501710),A301_2(190117310,193003010,193003110,193301810,193301910,193501810,193501910),B001_6(113006910),J0021(140032310),J0022(140032410),J022(140004810,140037510,140037610),J019_1(140004110),J020(140004310),J034(140007010),J038_2(140029850),J039(140008210),J041(140008410),J041_2(140039210),J042(140008510,140008670,140008770,140008810),J044_1(140009010),J038_1(140007710,140007810,140007910,140008170,140033770,140036710,140036810,140037910,140038010,140051010,140051110,180001210,140010210),J046(140010210),J0472(140010310),J0471(140051410),K545(150140010),K600(150148010,150148110,150275870),K602(150262910,150275710),K603(150266110,150266210,150301810),Acetylsalicylic_acid(610443053,620000065,620001952,620002629,620002761,620006661,620007816,620009301,610443049,610443052,610443054,610443056,610443058,621419201,621374801,621374901,621375001,621419401),Enoxaparin_sodium(620006786),Fondaparinux_sodium(620004874,620004875),Dalteparin_sodium(620002958,620002986,620006703,621699701,621701901,640407095,620002294,620002983,620002994,620002995,620002959,620004153,620004154,620005209,620006328,621757301,621671901,621673901,621757401,620812701,621702701,621673101,620002588,620007360,621994801,621994901,621995001,621995101),Heparin_calcium(643330002,643330007,643330003,620812201,620006720,620007487,620006721,621824801,620006722,620006723,620007488,621824901,620006724,621825101,621933401),Heparin_sodium(643330009,643330009,643330021,643330026,643330027,643330029,640450015,620003099,620003100,620004326,620004327,643330010,643330011,643330031,643330033,620003101,620008395,643330012,620006725,620006726,620006727,620008392,621825301,620006728,620006729,620006728,620006731,620006732,620006733,620007490,620007489,621825401,621825501,621825701,620006734,621825903,620006736,620006737,620006738,620007491,620008393,621825901,621826001,620006739,620006740,620006741,620006742,620007492,620008394,621826301),Warfarin_potassium(621938101,621940901,613330001,613330003,610450012,610460002,620000730,620000731,620002473,613330001,613330002,613330004,610463227,610462024,620002332,620002472,610463227,610463228,610462025),Adrenaline(642450005,642450164,620517902),Noradrenaline(642450071,642450165,620008384),Dopamine_hydrochloride(640461008,642110074,642110018,642110090,642110075,642110048,642110089,642110049,642110050,642110080,642110052,642110082,642110056,642110085,642110088,642110096,640431041,620001305,620002175,620003097,620003427,620003428,620003770,620004410,620005804,620005858,620008402,620008381,620009260,620244722,620244711,642110055,620003769,640461007,642110046,642110079,620002174,620008380,621399003,640461007,640461010,642110047,642110081,642110057,620003771,620002176,620008382,620245102,621958101,640461010,640461010,640461009,642110098,642110125,640421089,620003204,642110099,642110126,640421090,620003206,642110133,640407131,640433014,620002179,620003209,620003205,642110134,640407132,640433013,620002180,620003208,620003207,620004160,640462044,620003194,640462045,620003195,620004105),Dobutamine_hydrochloride(620006754,642110084,642110110,642110127,642110130,642110132,640406128,640406129,640406181,620002181,620003227,620004749,620005187,620007387,620007388,620007550,621365314,640407199,640463092,620003223,620005188,640407200,640463093,620003224,620005189,640462053,620003225,640462054,620003226,620004161),beniron(646340457,621151301,646340458,621151601,646340319,646340459,621151701,640453074,621449901),ganmaberin(646340322,646340323,646340481,646340328,646340329,646340463,621152101),guroberin(646340465,621152901,646340467,621153301,640453073,621450001),groburin(646340054,646340427,646340428,646340054,646340430,646340431,640450012,621157613,621157614,621157615,621157616,621157601,621157602),sanguroporu(646340341,620008826),ganmagado(646340435,620007259),Polyglobin(646340449,620004162,621758001,646340450,620004163,621758101,620004164,621758201),Venoglobulin(640421041,621159901,646340479,640421042,621160201,640421040,621160501,640463056,621490001),FOY(640461052,643990080,643990082,643990083,643990063,643990085,643990066,643990067,643990088,643990068,643990089,643990090,640453187,620002589,620003096,620003670,620003681,620003754,620005178,640461053,643990116,643990117,643990133,640406011,640406112,620001334,620004303),Nafamostat_mesilate(620003461,640453136,640453111,640453118,640453124,640453122,640453104,640453120,640453132,640453102,640453128,640453107,640453130,640453126,640453116,640453114,640463088,620007296,620001886,643990104,640453137,640453112,640453119,640453125,640453123,640453105,640453121,640453133,640453103,640453129,640453108,640453131,640453127,640453117,640453115,640453109,640463087,620007297,640453113,640453106,640453110,620000249,620000252,620000253,620000251,620007298,640451020,640451021),Ulinastatin(640406189,620005227,640406190,640422076,640422078,640433010,620005228,640406188,640422077,640422079,640433011,620005229,643990074,640406164,620006295,643990075,640406165,620006296,643990076,640406163,620006297,640406200,640406198),vancomycin_powder(616110042,620003894,620003895,620004465,620006663,620006791,620008047),vancomycin_liquid(646110044,640463091,640463090,640463102,640463043,640463079,620002907,620005694,620005695,620006696,620006792,640412114,620004644),arbekacin_sulfate(646110042,620004159,620004729,620008229,620008792,646110043,640422051,640422070,620003760,620004756,620004730,620008793,640421088,620004728,620007432),teicoplanin(640421065,620004709,620008212,620008213,620008532,620008531,620008533,620008534,620008535),dalfopristin(640462005),linezolid(610451000,640451010),Dioctyl_sodium_sulfosuccinate(612350034,612350040,620008725,620492501),Laxoberon(612350104,612350105,612350106,612350134,612350136,612350140,612350145,610406333,610407176,610453105,620003962,620004508,620005068,620006914,620007051,620007123,620008692,620009350,620488701,612350132,610422132,612350108,612350139,620006076,620007046,612350135,610407177,610407399,610412148,620004568,620004911,620007044,620490201,612350093,612350098,612350110,612350111,612350100,612350112,612350044,612350133,612350141,612350143,610412150,620005078,620005091,620005109,620006915,620006977,620007045,620007124,620007125,620490701,620491401,621977401,621993001,620491205),Magucolor(617210061,617210118),MaguP(610444096,617210100),nifrec(617990001,610433058,610444037,610444102,610444145,620007017,620008170,621201501,621201401,621360701,621827701),VIsiclear(620004869,621783701),J045(140009310,140009450,140009550,140009650,140009750,140009850,140009950,140010050,140010150,140023510,140023650,140023750,140023850,140023950,140024050,140024150,140024250,140024350,140030830,140030930,140031030,140031130,140031230,140031330,140031430,140031530,140031630,140031730,140039550,140039650,140039850),yuketsu(150224810,150247010,150254810,150286210,150286410,150286510,150224910,150286310),L002(150232610,150232710,150232810),L004(150232910),L008(150233410,150328210)'

register ./DPCPigUDF.jar;

define Exists jp.ac.u.tokyo.m.dpc.pig.udf.filter.Exists('$GFF');

define InnerGroup jp.ac.u.tokyo.m.dpc.pig.udf.eval.group.InnerGroup('$GFF');

B = load '/dpcdummydata' as (hospcd : chararray,ptid : chararray,dtdisc : chararray,dtadm : chararray,datakubun : chararray,jun_seqno : chararray,meiseqno : chararray,byouten : chararray,rececd : chararray,kaino : chararray,mei_mei : chararray,ryou : float,kitanni : chararray,meiten : float,meiyaku : float,meisai : float,enten : chararray,dekiten : float,dekihou : chararray,nyuseqno : int,sinnengetu : chararray,zissibi : chararray);

B1 = FOREACH B generate (chararray)hospcd,(chararray)ptid,(chararray)dtadm,rececd,zissibi,ryou,1 as cnt;

C1 = FILTER B1 by Exists(rececd);

Z1 = GROUP C1 by ($0,$1,$2) PARALLEL 35;

Z2 = FOREACH Z1 generate group, InnerGroup(C1, C1.$3);

Z3 = FOREACH Z2 generate group, Flatten(innergroup);

Z4 = FOREACH Z3 generate group.$0,group.$1,group.$2

,'Transfusion',MAX(innergroup::group_bag_yuketsu.cnt),SUM(innergroup::group_bag_yuketsu.ryou)

,'L002',MAX(innergroup::group_bag_L002.cnt),SUM(innergroup::group_bag_L002.ryou)

,'L004',MAX(innergroup::group_bag_L004.cnt),SUM(innergroup::group_bag_L004.ryou)

,'L008',MAX(innergroup::group_bag_L008.cnt),SUM(innergroup::group_bag_L008.ryou)

,'A300',MIN(innergroup::group_bag_A300.zissibi),MAX(innergroup::group_bag_A300.zissibi)

,'A301',MIN(innergroup::group_bag_A301.zissibi),MAX(innergroup::group_bag_A301.zissibi)

,'A301_2',MIN(innergroup::group_bag_A301_2.zissibi),MAX(innergroup::group_bag_A301_2.zissibi)

,'B001_6',MIN(innergroup::group_bag_B001_6.zissibi),MAX(innergroup::group_bag_B001_6.zissibi)

,'J0021',MIN(innergroup::group_bag_J0021.zissibi),MAX(innergroup::group_bag_J0021.zissibi)

,'J0022',MIN(innergroup::group_bag_J0022.zissibi),MAX(innergroup::group_bag_J0022.zissibi)

,'J022',MIN(innergroup::group_bag_J022.zissibi),MAX(innergroup::group_bag_J022.zissibi)

,'J019_1',MIN(innergroup::group_bag_J019_1.zissibi),MAX(innergroup::group_bag_J019_1.zissibi)

,'J020',MIN(innergroup::group_bag_J020.zissibi),MAX(innergroup::group_bag_J020.zissibi)

,'J034',MIN(innergroup::group_bag_J034.zissibi),MAX(innergroup::group_bag_J034.zissibi)

,'J038_2',MIN(innergroup::group_bag_J038_2.zissibi),MAX(innergroup::group_bag_J038_2.zissibi)

,'J039',MIN(innergroup::group_bag_J039.zissibi),MAX(innergroup::group_bag_J039.zissibi)

,'J041',MIN(innergroup::group_bag_J041.zissibi),MAX(innergroup::group_bag_J041.zissibi)

,'J041_2',MIN(innergroup::group_bag_J041_2.zissibi),MAX(innergroup::group_bag_J041_2.zissibi)

,'J042',MIN(innergroup::group_bag_J042.zissibi),MAX(innergroup::group_bag_J042.zissibi)

,'J044_1',MIN(innergroup::group_bag_J044_1.zissibi),MAX(innergroup::group_bag_J044_1.zissibi)

,'J045',MIN(innergroup::group_bag_J045.zissibi),MAX(innergroup::group_bag_J045.zissibi)

,'J038_1',MIN(innergroup::group_bag_J038_1.zissibi),MAX(innergroup::group_bag_J038_1.zissibi)

,'J046',MIN(innergroup::group_bag_J046.zissibi),MAX(innergroup::group_bag_J046.zissibi)

,'J0472',MIN(innergroup::group_bag_J0472.zissibi),MAX(innergroup::group_bag_J0472.zissibi)

,'J0471',MIN(innergroup::group_bag_J0471.zissibi),MAX(innergroup::group_bag_J0471.zissibi)

,'K545',MIN(innergroup::group_bag_K545.zissibi),MAX(innergroup::group_bag_K545.zissibi)

,'K600',MIN(innergroup::group_bag_K600.zissibi),MAX(innergroup::group_bag_K600.zissibi)

,'K602',MIN(innergroup::group_bag_K602.zissibi),MAX(innergroup::group_bag_K602.zissibi)

,'K603',MIN(innergroup::group_bag_K603.zissibi),MAX(innergroup::group_bag_K603.zissibi)

'Acetylsalicylic_acid',MIN(innergroup::group_bag_Acetylsalicylic_acid.zissibi),MAX(innergroup::group_bag_Acetylsalicylic_acid.zissibi)

'Enoxaparin_sodium',MIN(innergroup::group_bag_Enoxaparin_sodium.zissibi),MAX(innergroup::group_bag_Enoxaparin_sodium.zissibi)

'Fondaparinux_sodium',MIN(innergroup::group_bag_Fondaparinux_sodium.zissibi),MAX(innergroup::group_bag_Fondaparinux_sodium.zissibi)

'Dalteparin_sodium',MIN(innergroup::group_bag_Dalteparin_sodium.zissibi),MAX(innergroup::group_bag_Dalteparin_sodium.zissibi)

'Heparin_calcium',MIN(innergroup::group_bag_Heparin_calcium.zissibi),MAX(innergroup::group_bag_Heparin_calcium.zissibi)

'Heparin_sodium',MIN(innergroup::group_bag_Heparin_sodium.zissibi),MAX(innergroup::group_bag_Heparin_sodium.zissibi)

'Warfarin_potassium',MIN(innergroup::group_bag_Warfarin_potassium.zissibi),MAX(innergroup::group_bag_Warfarin_potassium.zissibi)

'Adrenaline',MIN(innergroup::group_bag_Adrenaline.zissibi),MAX(innergroup::group_bag_Adrenaline.zissibi)

'Noradrenaline',MIN(innergroup::group_bag_Noradrenaline.zissibi),MAX(innergroup::group_bag_Noradrenaline.zissibi)

'Dopamine_hydrochloride',MIN(innergroup::group_bag_Dopamine_hydrochloride.zissibi),MAX(innergroup::group_bag_Dopamine_hydrochloride.zissibi)

'Dobutamine_hydrochloride',MIN(innergroup::group_bag_Dobutamine_hydrochloride.zissibi),MAX(innergroup::group_bag_Dobutamine_hydrochloride.zissibi)

'beniron',MIN(innergroup::group_bag_beniron.zissibi),MAX(innergroup::group_bag_beniron.zissibi)

'ganmaberin',MIN(innergroup::group_bag_ganmaberin.zissibi),MAX(innergroup::group_bag_ganmaberin.zissibi)

'guroberin',MIN(innergroup::group_bag_guroberin.zissibi),MAX(innergroup::group_bag_guroberin.zissibi)

'groburin',MIN(innergroup::group_bag_groburin.zissibi),MAX(innergroup::group_bag_groburin.zissibi)

'sanguroporu',MIN(innergroup::group_bag_sanguroporu.zissibi),MAX(innergroup::group_bag_sanguroporu.zissibi)

'ganmagado',MIN(innergroup::group_bag_ganmagado.zissibi),MAX(innergroup::group_bag_ganmagado.zissibi)

'Polyglobin',MIN(innergroup::group_bag_Polyglobin.zissibi),MAX(innergroup::group_bag_Polyglobin.zissibi)

'Venoglobulin',MIN(innergroup::group_bag_Venoglobulin.zissibi),MAX(innergroup::group_bag_Venoglobulin.zissibi)

'FOY',MIN(innergroup::group_bag_FOY.zissibi),MAX(innergroup::group_bag_FOY.zissibi)

'Nafamostat_mesilate',MIN(innergroup::group_bag_Nafamostat_mesilate.zissibi),MAX(innergroup::group_bag_Nafamostat_mesilate.zissibi)

'Ulinastatin',MIN(innergroup::group_bag_Ulinastatin.zissibi),MAX(innergroup::group_bag_Ulinastatin.zissibi)

'vancomycin_powder',MIN(innergroup::group_bag_vancomycin_powder.zissibi),MAX(innergroup::group_bag_vancomycin_powder.zissibi)

'vancomycin_liquid',MIN(innergroup::group_bag_vancomycin_liquid.zissibi),MAX(innergroup::group_bag_vancomycin_liquid.zissibi)

'arbekacin_sulfate',MIN(innergroup::group_bag_arbekacin_sulfate.zissibi),MAX(innergroup::group_bag_arbekacin_sulfate.zissibi)

'teicoplanin',MIN(innergroup::group_bag_teicoplanin.zissibi),MAX(innergroup::group_bag_teicoplanin.zissibi)

'dalfopristin',MIN(innergroup::group_bag_dalfopristin.zissibi),MAX(innergroup::group_bag_dalfopristin.zissibi)

'linezolid',MIN(innergroup::group_bag_linezolid.zissibi),MAX(innergroup::group_bag_linezolid.zissibi)

'Dioctyl_sodium_sulfosuccinate',MIN(innergroup::group_bag_Dioctyl_sodium_sulfosuccinate.zissibi),MAX(innergroup::group_bag_Dioctyl_sodium_sulfosuccinate.zissibi)

'Laxoberon',MIN(innergroup::group_bag_Laxoberon.zissibi),MAX(innergroup::group_bag_Laxoberon.zissibi)

'Magucolor',MIN(innergroup::group_bag_Magucolor.zissibi),MAX(innergroup::group_bag_Magucolor.zissibi)

'MaguP',MIN(innergroup::group_bag_MaguP.zissibi),MAX(innergroup::group_bag_MaguP.zissibi)

'nifrec',MIN(innergroup::group_bag_nifrec.zissibi),MAX(innergroup::group_bag_nifrec.zissibi)

'VIsiclear',MIN(innergroup::group_bag_VIsiclear.zissibi),MAX(innergroup::group_bag_VIsiclear.zissibi)

;

Store Z4 into 'fromf2' using jp.ac.u.tokyo.m.dpc.pig.udf.store.StoreDataWith Schema();
